# What effect does physician "profiling" have on inpatient physician satisfaction and hospital length of stay?

**DOI:** 10.1186/1472-6963-6-45

**Published:** 2006-04-04

**Authors:** Judith K Zemencuk, Timothy P Hofer, Rodney A Hayward, Richard H Moseley, Sanjay Saint

**Affiliations:** 1 Health Services Research & Development Field Program, Ann Arbor VA Center for Practice Management and Outcomes Research, Ann Arbor, Michigan, USA; 2 Medical Service, Ann Arbor VA Medical Center and Department of Internal Medicine, The University of Michigan Medical School, Ann Arbor, Michigan, USA

## Abstract

**Background:**

2002 marked the first time that the rate of hospital spending in the United States outpaced the overall health care spending rate of growth since 1991. As hospital spending continues to grow and as reimbursement for hospital expenses has moved towards the prospective payment system, there is still increasing pressure to reduce costs. Hospitals have a major incentive to decrease resource utilization, including hospital length of stay. We evaluated whether physician profiling affects physician satisfaction and hospital length of stay, and assessed physicians' views concerning hospital cost containment and the quality of care they provide.

**Methods:**

To determine if physician profiling affects hospital length of stay and/or physician satisfaction, we used quasi-experimental with before-versus-after and intervention-versus-control comparisons of length of stay data collected at an intervention and six control hospitals. Intervention hospital physicians were informed their length of stay would be compared to their peers and were given a questionnaire assessing their experience.

**Results:**

Nearly half of attending pre-profiled physicians felt negative about the possibility of being profiled, while less than one-third of profiled physicians reported feeling negative about having been profiled. Nearly all physicians greatly enjoyed their ward month. Length of stay at the profiled site decreased by an additional 1/3 of a day in the profiling year, compared to the non-profiled sites (p < 0.001).

**Conclusion:**

A relatively non-instrusive profiling intervention modestly reduced length of stay without adversely affecting physician satisfaction.

## Background

The cost of providing health care to hospitalized patients is enormous. While recent data on national trends in hospitalization show that the average length of a hospital stay in the United States has declined from 7.3 days in 1980 to 4.9 days in 2000 [1], spending on hospital care increased 9.5% in 2002 to $486.5 billion, marking the first time the rate of hospital spending outpaced the overall health care spending rate of growth since 1991 [2]. The Department of Veterans Affairs ("VA") operates one of the nation's largest healthcare systems, providing health care for eligible veterans. Facilities operated by the VA traditionally have had significantly longer lengths of stay than those of the private sector [3-5] and, while there is evidence that the gap in length of stay between VA facilities and the private sector may be narrowing [6], there is still increasing pressure to reduce costs. As hospital spending continues to grow and as reimbursement for hospital expenses has moved towards the prospective payment system, hospitals have a major incentive to decrease resource utilization, including hospital length of stay. Various methods have been proposed to control hospital costs and decrease inpatient length of stay, including utilization review, critical pathways, physician education, and the use of hospitalists [7-16].

Physician profiling is another process that has been used in an attempt to constrain spending and reduce inpatient length of stay [17-19]. Physician profiling is the practice whereby the practice pattern of a single physician or a group is expressed as some measure of the use of resources and/or length of stay during a defined period for the population served. The resulting profile is then compared with a norm that is either based on practice (such as profiles of other physicians) or on standards (such as practice guidelines) [20]. According to results of the 2000–2001 Community Tracking Study Physician Survey from The Center for Studying Health System Change, more than a third of physicians have reported that practice profiles had influenced their practice of medicine [21]. In addition, results indicate that physicians may view profiling in a more positive light than before. Nearly half of physicians surveyed, however, still maintained a mixed or negative view of physician profiling [21]. Nevertheless, some believe that comparing a physician's resource consumption level to a benchmark figure may encourage the physician to conserve resources to avoid classification as a resource consumption outlier.

Physician profiling has had mixed success [17-19,21-23]. We thus evaluated whether physician profiling affects physician satisfaction and hospital length of stay, while assessing physicians' views concerning hospital cost containment and the quality of care they provide.

## Methods

### Study site and participants

The study was conducted at a Veterans Affairs Medical Center hospital affiliated with a major university medical school (the "intervention site") in the Midwestern United States. The Veterans Affairs' Institutional Review Board and the Research & Development Committee and the Subcommittee on Human Studies approved the study. All general medicine physicians who attended at the intervention site during the period July 1, 1999 through June 30, 2001 (68 senior residents and 74 attending physicians) were included in the study.

### Study period

The pre-profiling and profiling periods of the study were conducted in two phases over a two-year period. The pre-profiling period took place from July 1, 1999 through June 30, 2000. The profiling period included the period from July 1, 2000 through June 30, 2001. During both phases of the study, all attending physicians and senior residents on all four medical services of the intervention site were sent a short, self-administered questionnaire at the end of their ward month intended to measure their overall satisfaction during that month. During the profiling period, all attending physicians on the four medical services at the intervention site were informed that they would be profiled. At the beginning of each ward month, a brief meeting between the Chief of the Medical Service and the four attending physicians assigned to each medical team in which expectations regarding attending supervision, teaching, and documentation are discussed is held. Throughout the profiling period, this standard orientation included the following statement read by the Service Chief:

"As you may know, the VA, at a national level, is exploring various methods of reducing patient length of stay. One proposed method involves the profiling of attending physicians. All medical ward services will be profiled this month. By profiling, I mean that your patients' lengths of stay will be compared to the patient length of stay of other attendings. We will, of course, use patient risk-adjustment methods when comparing your team's performance to other teams' data. At the end of the month, you and your team will then be given a short questionnaire in which your attitudes about profiling will be assessed. All of your responses will be kept confidential."

Each attending physician was also sent a letter from the Service Chief reminding him or her that they were being profiled.

### Survey instruments

We developed three self-administered questionnaires for this study: one for senior residents (8 questions), one for attending physicians administered during the pre-profiling phase (14 questions), and a second for physicians who attended during the profiling phase (23 questions). The questionnaires were largely comprised of questions conceived of by the study authors. The questions are specific to content domains identified by the study authors as important and appropriate to the study theme. We reproduce the exact wording of all key questions in the results that follow. The senior residents' questionnaire was designed to assess: 1) their satisfaction during their ward month; 2) their perception of the extent to which their attending physicians were involved in patient care decisions; and 3) the degree of autonomy in making patient care decisions allowed them by their attending physician. The same senior resident questionnaire was used during both the pre-profiling and profiling phases. Attending physician questionnaires administered during the pre-profiling phase were designed to assess: 1) their satisfaction during their ward month; 2) how they thought they would react to being profiled; 3) their feelings concerning economic aspects of patient care [24]; and 4) their perception of the quality of care they provided. Questionnaires mailed to attending physicians during the profiling phase differed from the pre-profiling attending physician questionnaire in that respondents were also asked to indicate if being profiled caused them to decrease the ordering of tests and/or procedures, decrease their patients' lengths of stay, whether they felt pressured to discharge a patient from the hospital prematurely, and whether they were more involved than usual in the care of their patients.

### Medical information systems data

We used the Department of Veterans Affairs' Patient Treatment File for July 1, 1998 through June 30, 2002. The Patient Treatment File contains a standardized hospital discharge abstract describing each patient's demographic (e.g., age, sex, race) and clinical characteristics (ICD-9-CM diagnosis and procedure codes). Diagnoses include the primary diagnosis and up to nine secondary diagnoses. We retrieved Patient Treatment File data for the intervention site for the pre-profiling phase (July 1, 1999 through June 30, 2000) and the profiling phase (July 1, 2000 through June 30, 2001). For the purpose of comparison to the study period, we obtained data for the year preceding the pre-profiling phase (July 1, 1998 through June 30, 1999) and for the year following the profiling phase (July 1, 2001 through June 30, 2002). Also for comparison purposes, we analyzed data for six control hospitals in the same VA hospital network as the intervention site. The total study sample included 9,307, 9,250, 9,319, and 9,633 admissions for the years July 1, 1998 through June 30, 2002, respectively. At the intervention site, the pre pre-profiling year (July 1, 1998 through June 30, 1999), the pre-profiling phase (July 1, 1999 through June 30, 2000), the profiling phase (July 1, 2000 through June 30, 2001), and the post-profiling year (July 1, 2001 through June 30, 2002) included 2,186, 1,982, 2,145, and 2,162 admissions, respectively.

### Data analysis

#### Survey data

We tested for statistically significant differences in physician responses between phases using the Student's *t *test for continuous variables and chi square analysis for categorical variables, with a criterion of p < 0.05. We next tested for statistically significant differences using logistic regression to control for possible confounding effects related to physician characteristics, such as years since graduation from medical school, type of work planning to pursue after completion of residency (primarily outpatient- or inpatient-based general internal medicine, or subspecialty fellowship), and sex of senior residents. For attending physicians, independent variables included years since graduation from medical school, years attending at the hospital, primary specialty, and subspecialty. For the 5-item Likert scale response sets that ranged from "strongly disagree" to "strongly agree," from "very positive" to "very negative," and from "a great deal" to "not at all," we trichotomized responses (e.g., "disagree/strongly disagree," "neither agree nor disagree," and "agree/strongly agree").

#### Medical information system data

The main dependent variable of interest was hospital length of stay. The principle independent variable of interest was study phase (pre-profiling versus profiling). We tested for a change in length of stay associated with profiling occurring above and beyond the temporal trends in length of stay. The distribution of hospital length of stay is heavily skewed with a long right tail and some very high length of stay outliers. Such extreme outliers usually represent medically unique patients and we thus truncated length-of-stay outliers, defined as those patients with length of stay greater than 2 standard deviations above the mean length of stay [8]. In addition, we tested for differences in length of stay between the pre-profiling and profiling phases using robust regression, which uses a pseudovalues method that does not assume a normal distribution of the dependent variable [25,26]. Comorbidity was measured using a validated approach proposed by Elixhauser and colleagues [27]. This approach uses secondary ICD-9 codes and considers 30 diagnoses. Algorithms exclude conditions that are related to the admission diagnosis and are unlikely to represent complications of care [27]. We also adjusted for patient demographics (age, marital status, race, and sex), discharge destination, and primary diagnosis.

## Results

### Survey results

#### Physician characteristics

The response rate was 84% for senior residents (37 of 44) in the pre-profiling period and 80% (31 of 39) during the profiling period, and 90% for attending physicians (38 of 42) in the pre-profiling period and 90% (36 of 40) during the profiling period. Approximately 35% of attending physicians were women, as were 42% of senior residents. Attending physicians had attended at the VA Medical Center for an average of 7.4 years. As shown in Table 1, there were no significant differences in senior resident or attending attributes between phases.

#### Physician views on cost containment

The majority of both groups of attending physicians disagreed that it is "unfair to ask physicians to be cost-conscious and still keep the welfare of their patients foremost in their minds" (65.8% and 77.8% of pre-profiling and profiling attending physicians, respectively). Similarly, most attending physicians agreed that "trying to contain costs is the responsibility of every physician," with 81.1% and 86.1% of pre-profiling and profiling attending physicians, respectively, agreeing. There were no statistically significant differences between groups.

#### Physician satisfaction

During both the pre-profiling and profiling time periods, over 80% of all physicians, both attendings and senior residents reported greatly enjoying their ward month (Table 2).

#### Physician reports on quality of care

As shown in Table 2, the vast majority of both the pre-profiling and profiling groups of senior residents agreed that they provided extremely high quality care to their patients during their ward months. In addition, the vast majority of both groups of attending physicians agreed that they were "very involved in the day-to-day care" of their patients and that their ward team "provided extremely high quality care." Nearly half (45.7%) of profiled physicians, however, reported that they were more involved than usual in the care of the patients on their service during their profiling month. There was a non-significant trend in the difference between responses of the pre-profiling and profiling senior residents when asked to indicate their agreement with the statement "My attending was very involved in all important patient care decisions" (p = .054). Interestingly, more pre-profiled senior residents (62%) than senior residents in the profiled group (39%) agreed with this statement.

#### Physician views of profiling

There was a signficant difference between pre-profiling and profiling attending physicians regarding their feelings about being profiled (Table 2). Nearly half of pre-profiling attending physicians indicated that they would feel "negative" about the possibility of being profiled, while less than one-third of profiled attending physicians reported feeling "negative" about being profiled during their ward month (p = .037). While 34.2% of pre-profiling attending physicians agreed that being profiled "will cause most physicians to discharge some patients earlier than they would have normally," only 8.3% of profiled attending physicians agreed with this statement (p = .017).

As shown in Table 2, several survey questions were asked only of profiled attending physicians. Very few (14%) of profiled physicians indicated that they did "try to decrease [their] patients' lengths of stay" and none agreed that "being 'profiled' caused me to discharge some patients earlier than I would have normally." A similar percentage (11%) indicated that they did "try to decrease ordering of tests and procedures," while none, again, agreed that "being 'profiled' caused me to decrease the ordering of some tests and/or procedures."

### Length of stay results

On average, compared to the other network hospitals, the intervention site experienced longer average lengths of stay (0.66 days, CI = 0.49 to 0.83, p < .001) over the four-year period that includes the year preceding the pre-profiling year (July 1, 1998 through June 30, 1999) and the year following the profiling year (July 1, 2001 through June 30, 2002). There was, however, a significant trend of decreasing length of stay during this period (-0.05 days, CI = -0.08 to -0.02, p = .001) that varied by site. The length of stay decrease was greater at the intervention site (-0.27 days, CI = -0.32 to -0.21; p < .001) relative to the non-profiled sites.

During the profiling year, the average length of stay at the non-profiled sites was 5.8 days, while it was 6.1 days at the intervention site (p = 0.01). The intervention site, however, experienced an additional 0.32 day reduction in length of stay (CI = -0.49 to -0.16; p < .001), relative to the other network hospitals during the profiling period.

The data revealed that from the profiling year to the post-profiling year, there was no significant change in length of stay at the intervention site (-0.07 days, CI = -0.23 to 0.09, p = .43), while the other sites experienced a 0.23 day reduction (CI = -0.32 to -0.15, p < .001). Adjusting for casemix did not have a substantial effect on our findings.

## Discussion

Inpatient length of stay is generally the single greatest factor contributing to overall hospital costs [28]. The average length of a hospital stay has declined over the past decade; however, hospitals still face pressure to reduce costs by restricting length of stay for hospitalized patients. While there are significant geographic variations in the use of inpatient hospital services in the Veterans Health Administration [29], lengths of stay at VA hospitals have historically exceeded those of community hospitals [30,31]. Recent analyses, however, suggest that the disparity in length of stay between VA facilities and non-federal hospitals is lessening [32].

The decrease in inpatient length of stay may reflect, among other factors, the recent use of more aggressive utilization management techniques, the shifting of care to outpatient venues [33], and the hospitalist model of care [7,9,11-13]. Since 1995, Veterans Health Administration facilities have undergone extensive restructuring and realignment in order to improve healthcare service delivery and administrative operations [34]. However, as the veteran population ages and new veterans enter the Veterans Health Administration system, the challenge of increasing efficiency will become even more important. All health professionals, however, are challenged to reduce unnecessary stays and services without sacrificing quality of care. As hospitals face pressures to contain costs, it is important to understand the usefulness of various methods of reducing resource utilization, including patient length of stay.

Previous studies evaluating the effects of physician profiling are instructive in placing our results in proper context. Ross et al. [18] undertook a study in which physicians were sent profile reports that included information regarding the number of patients and lengths of stay for each DRG the profiled physician had admitted during two-month periods. Each profiled physician was also sent a letter that included benchmark figures for lengths of stay for the critical pathways that were in use at the study facility. The results suggest that profiling had a strong influence on physicians' behaviours, resulting in a decrease in average length of stay. Balas and colleagues [23] performed a multilevel meta-analysis designed to assess the clinical effect of peer-comparison feedback intervention (profiles) in changing practice patterns. They found physician profiling to have a statistically significant though minimal effect on the utilization of clinical procedures. Evans and colleagues [17] analyzed the effectiveness of one hospital's introduction of physician length of stay profiling. Controlling for physician, DRG, and patient severity level, data for patients treated by 400 physicians over 42 months were analyzed, including both pre-profiling and post-profiling periods. There was a significant increase in the percentage of physicians who achieved the length of stay benchmark after the introduction of profiling.

It is also important when interpreting the findings of the current study to consider several potential limitations. First, since the intervention site consisted of a single VA medical center, some may question the external validity of our findings. However, our intervention was not "VA-specific" in that a similar type of intervention could readily be implemented in virtually any type of hospital. Second, the cause of the significant decrease in length of stay may have less to do with profiling and more to do with other issues. Specifically, as Evans and colleagues [17] have pointed out, results from profiling studies may also reflect the influence of other factors, such as physicians substituting more procedures for the reduction in length of stay in response to concerns such as exposure to legal liability. Finally, since we did not conduct a randomized trial, confounding variables – such as case-mix changes or co-interventions – could have influenced our results. We did, however, adjust for case-mix and temporal trends at other institutions in our statistical analyses.

## Conclusion

In this study, we describe an investigation of physician profiling as a method of decreasing inpatient length of stay. While the majority of attending physicians agreed that trying to contain costs is the responsibility of every physician, nearly half expressed negative feelings toward being profiled prior to the intervention. A much smaller proportion of physicians who were profiled, however, viewed the process negatively; being profiled had no apparent effect on satisfaction during profiling. Additionally, profiling of attending physicians appeared to have no significant effect on senior residents' perception of the degree of involvement by their attending physicians between pre- and profiling years. Finally, the majority of both senior resident and attending physicians reported that they provided extremely high quality care. Although survey responses suggested that profiling would not cause physicians to discharge patients early, after taking into account the background trend and relative to the non-profiled sites, length of stay at the profiled site decreased by one-third of a day in the profiling year.

Despite limitations discussed previously, our results raise several important questions. While anticipation of being profiled was viewed as negative by a large number of attending physicians, most of those who were profiled did not view the process negatively. It may be that the unknown (i.e., the possibility of being profiled) is more threatening than the actuality of being profiled. In addition, while it is difficult to determine to what extent the reduction in length of stay was due to the profiling, it is noteworthy that physicians' reported intent that being profiled would not cause them to decrease patient length of stay differed from the results (i.e., a reduction in length of stay while being profiled). Our findings indicate that profiling may be an effective method of decreasing length of stay without affecting physician satisfaction.

## Competing interests

The author(s) declare that they have no competing interests.

## Authors' contributions

All authors developed the study design and protocol. All authors were responsible for implementing the study, analyzing the results, and/or contributing to drafting the report and approved final manuscript.

## Pre-publication history

The pre-publication history for this paper can be accessed here:



## Figures and Tables

**Table 1:** Physician Characteristics (N = 142)

	Pre-Profiling Phase* n = 38	Profiling Phase* n = 36
***Attending Physicians (n = 74)***		
		
Mean years since graduation from medical school (s.d.)	19.4 (9.2)	17.9 (8.5)
Mean years attending at the VA Medical Center (s.d)	7.1 (6.6)	7.8 (7.0)
Female, % (n)	33 (12)	37 (13)
Specialty:< % (n)		
General internal medicine	36.1 (13)	40 (14)
Internal medicine subspecialty	63.9 (23)	60 (21)
Subspecialty (total may differ from sum due to rounding error):		
Endocrinology	7.9 (3)	5.6 (2)
Geriatrics	18.4 (7)	16.7 (6)
Hypertension	0.0 (0)	2.8 (1)
Infectious diseases	10.5 (4)	13.9 (5)
Nephrology	2.6 (1)	0.0 (0)
Nuclear medicine	7.9 (3)	2.8 (1)
Pulmonary critical care	2.6 (1)	0.0 (0)
Rheumatology	10.5 (4)	11.1 (4)
None	36.8 (14)	44.4 (16)
	Pre-Profiling Phase* n = 37	Profiling Phase* n = 31
Senior Resident Physicians (n = 68)		
		
Mean years since graduation from medical school(s.d.)	3.6 (1.8)	3.2 (1.1)
Female :< % (n)	47.2 (17)	35.5 (11)
Work pursuing after residency: :< % (n)		
Primarily outpatient-based general internal medicine	25.0 (9)	12.9 (4)
Primarily inpatient-based general internal medicine	8.3 (3)	6.5 (2)
Subspecialty fellowship	66.7 (24)	80.6 (25)

* None of the differences between pre-profiling and profiling period physicians were statistically significant.

**Table 2:** Physician Survey Responses (N = 142)

	***Pre-Profiled Attendings n (%) n = 38***	***Profiled Attendings n (%) n = 36***	***Pre-Profiled Senior Residents n (%) n = 37***	***Profiled Senior Residents n (%) n = 31***
**ENJOYMENT OF WARD MONTH – AGREED THAT:**				
"I greatly enjoyed my most recent ward month"	34 (89)	32 (89)	31 (84)	25 (81)
**QUALITY OF CARE – AGREED THAT:**				
"I was very involved in the day-to-day care of the patients on my service."	34 (89)	30 (83)		
"My attending was very involved in all important patient care decisions."			23 (62)	12 (39)
"I allowed my residents considerable autonomy in patient care decisions."	38 (100)	35 (97)		
"My attending allowed me considerable autonomy in making patient care decisions."			32 (86)	29 (94)
"My ward team provided extremely high quality care to our patients."	37 (97)	33 (92)		
"I provided extremely high quality care to my patients."			33 (89)	28 (90)
**FEELINGS ABOUT PROFILING**				
"How do you think you would feel about being 'profiled?'?" OR "How did you feel about being 'profiled?"'				
Positive	11 (29)	6 (17)		
Indifferent	10 (26)	20 (56)		
Negative	17 (45)	10 (28)*		
**PERCEPTION OF PROFILING EFFECT ON PATIENT CARE – AGREED THAT:**				
"In general, being 'profiled' will cause most physicians to discharge some patients earlier than they would have normally."	13 (34)	3 (8)*		
"In general, being 'profiled' will cause most physicians to decrease the ordering of some tests and/or procedures."	17 (46)	5 (14)*		
**PROFILING EFFECT (QUESTIONS ASKED ONLY OF PHYSICIANS WHO WERE PROFILED)**				
Reported that they tried "to become more involved than usual in the care of the patients on [their] service during their profiling month.		16 (46)		
Agreed that "being 'profiled' caused me to discharge some patients earlier than I would have normally."		0 (0)		
Agreed that "I sometimes felt pressured to discharge a patient from the hospital prematurely."		2 (6)		
"Did you try to decrease your patients' lengths' of stay?" Responded "Yes:"		5 (14)		
Agreed that "being 'profiled' caused me to decrease the ordering of some tests and/or procedures."		0 (0)		
Agreed that "I sometimes felt pressured not to use certain ancillary tests/services."		0 (0)		
"Did you try to decrease ordering of tests and procedures?" Responded "Yes:"		4 (11)		

*p <.05 for differences between physician groups
